# Urine Discoloration After Voiding in a Boy With Ulcerative Colitis Using Mesalamine

**DOI:** 10.1097/PG9.0000000000000167

**Published:** 2022-01-24

**Authors:** Dagmar Zeef, Tim de Meij, Arend Bökenkamp

**Affiliations:** From the * Department of Pediatric Gastroenterology, Amsterdam University Medical Center, Amsterdam, the Netherlands; † Department of Pediatric Nephrology, Amsterdam University Medical Center, Amsterdam, the Netherlands.

A 12-year-old boy with ulcerative colitis noticed red-brown urine since several days. He did not have any other complaints, in particular, no abdominal pain, fever, or dysuria. The boy noticed the colorization only when he urinated in the toilet at home and this phenomenon was most pronounced after the toiled had been cleaned. The ulcerative colitis was clinically in remission with monotherapy mesalamine. On physical examination, no abnormalities were found. The urine collected at presentation was colored yellow. Urine analysis was performed, which was negative for leucocyturia, hematuria, or proteinuria.

The differential diagnosis of red-brown colored urine in ulcerative colitis patients includes hematuria due to nephrocalcinosis (1), urinary tract infection, and interstitial nephritis as an adverse effect of mesalamine. In the absence of hematuria, urine discoloration can be caused by ingestion of specific foods (e.g., beetroot), drugs (e.g., rifampicin), and metabolic products as bile pigments, myoglobin, and porphyrins (2). In our patient, fresh voided urine was yellow. With the suspicion of a chemical reaction between mesalamine in the urine and bleach, we asked the boy to urinate in a bleach-cleaned toilet. Discoloration occurred (Fig. 1 and 2). The discoloration did not appear when one of his parents urinated in the same bleach-cleaned toilet. While discoloration of urine by a chemical reaction between mesalamine and sodium hypochlorite bleach has been widely reported in online patient forums, we only found 2 related case reports (3).

**FIGURE 1.**
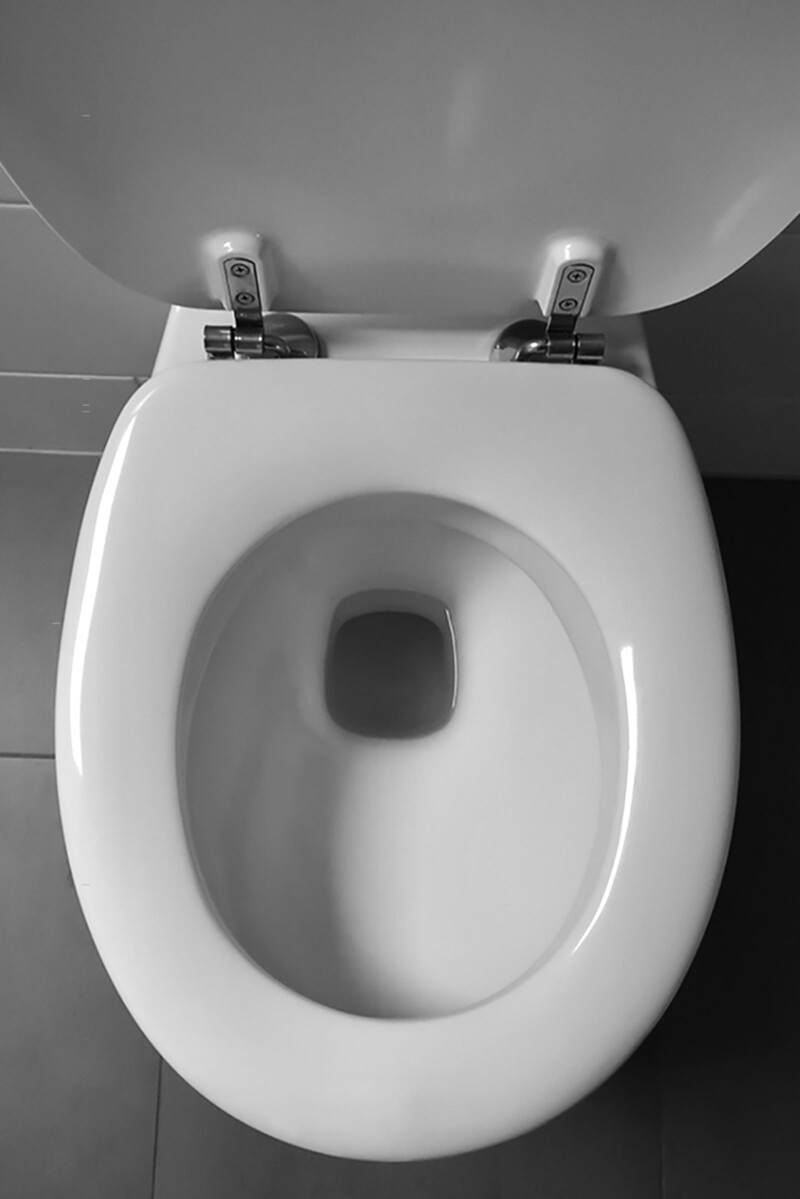
Toilet cleaned with bleach containing sodium hypochlorite.

**FIGURE 2.**
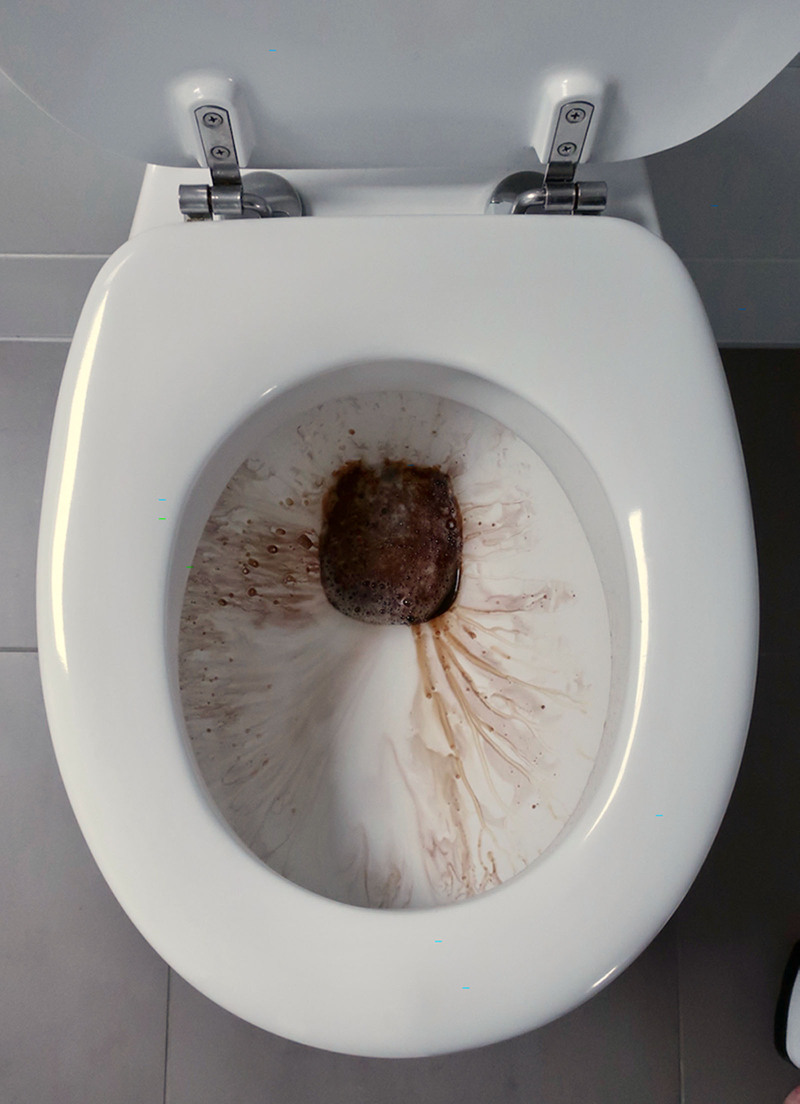
Discoloration of urine after contact with the freshly cleaned toilet.

The process leading to discoloration of urine after contact with bleach is not fully understood. Mesalamine and its metabolite, *N*-acetyl-5-aminosalicylic acid, have structural similarity to methyldopa, which is metabolized to melanin-like compounds. In an alkaline environment (the pH of bleach is 11–13), polymerization of these melanin-like metabolites causes a brownish/red discoloration of urine after methyldopa ingestion. Smeets et al suggest a similar reaction with mesalamine leading to polymerization of melanin-like metabolites in a highly alkaline environment (3). Alternatively, discoloration on contact with bleach could be due to an azo-dye, which is present in the chemical structure of mesalamine.

Our case illustrates that a drug widely prescribed in IBD can lead to a clinical presentation suspected of hematuria. Clues to the diagnosis are a normal urine color before contact with the toilet and the absence of erythrocytes in the urine sediment. Recognition of the phenomenon that bleach in the toilet can lead to discolored urine with mesalazine use avoids costly additional diagnostic tests and can prevent patient distress.

## ACKNOWLEDGMENTS

D.Z. contributed to the case design, drafting of the article, and literature research. A.B. contributed to the revision of the article for important intellectual content. T.d.M. contributed to the critical revision of the article for important intellectual content.
